# Supplementary material for: Arabidopsis ERF012 Is a Versatile Regulator of Plant Growth, Development and Abiotic Stress Responses
Source: Int J Mol Sci. 2022 Jun 20;23(12):6841. doi: 10.3390/ijms23126841 (PMC9224505; doi:10.3390/ijms23126841)
Supplement: Supplementary file 1 [file ijms-23-06841-s001.zip › ijms-1737361-supplementary.pdf]

Supplementary Figure S1

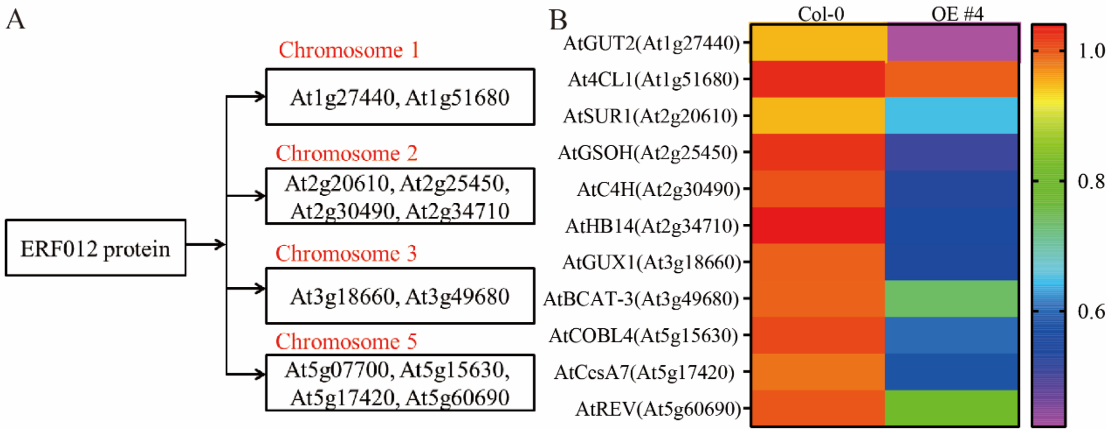

Supplementary Figure S1. ERF012 regulates the the genes involved cell wall formation. (A) The genes were were predicted to be the targets of ERF012 in the website (https://bar.utoronto.ca/eplant/). (B) The heatmap shows the expression of the ERF012 target genes in 12-day-old Col-0 and overexpression *ERF012* line (OE #4). Values represent means ± SD, n=3 pools, with about 20 plant roots per pool.

Supplement Figure S2

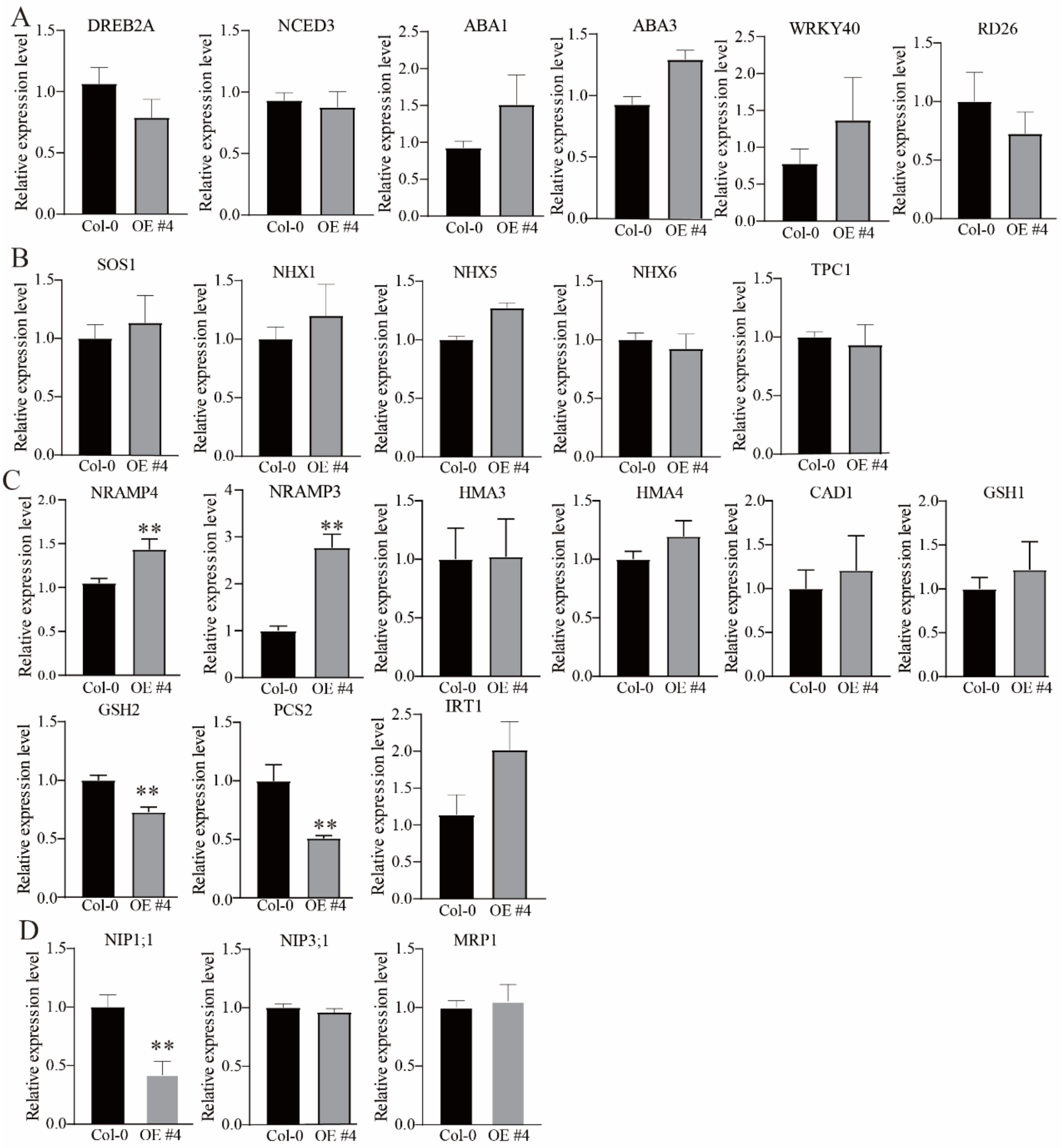

Supplementary Figure S2. ERF012 regulates the expression of the genes involved in drought, NaCl, Cd, As. (A-D) The expression of the genes involved in drought, NaCl, Cd, As stress in 12-day-old Col-0 and overexpression *ERF012* line. Values represent means ± SD, n=3 pools, with about 20 plant roots per pool. Asterisks indicate significant differences between different treatments: Student's *t*-test: **, $P < 0.01$.

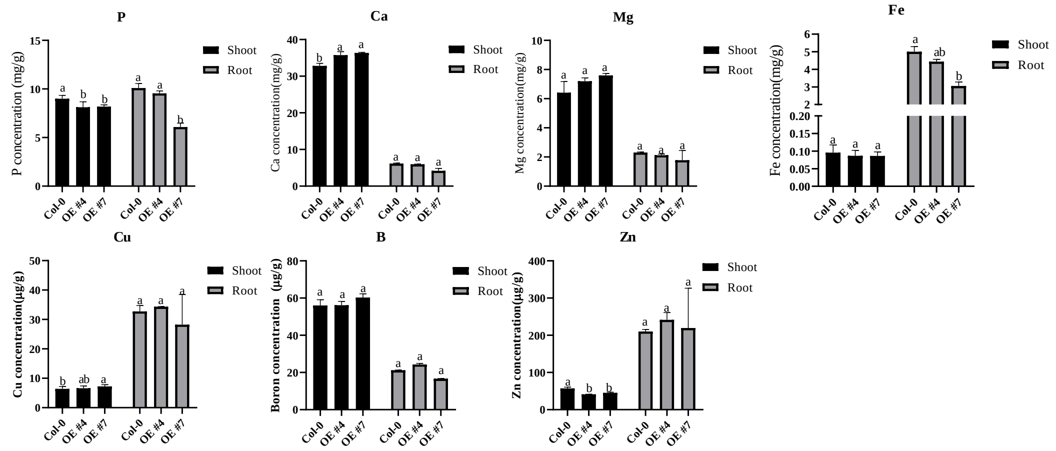

Supplement Figure S3: The nutrition in Col-0 and *ERF012* OE lines. The Col-0 and *ERF012* OE lines were grown in hydropic solution for 35 d. The shoots and roots were harvested and dried for nutrition detection. Values represent means ± SD, n=3. Letters indicate significant differences between different plants: Ducan's test (p<0.05).

Supplement Table S1. The specific primers were used in the paper.

Use	Primers (5'->3')
RT-qAtERF012-F	GTCGGCGTTGATGGATTTGGAGT
RT-qAtERF012-R	CTTGCTCTGAGCTGTTCTTGAGTATAGATC
RT-qAtACS7-F	GCTCGAACCATTTCCAGATAAC
RT-qAtACS7-R	AAGTGAATATTCTTGCGTACGC
RT-qAtACS11-F	CAGTTGTTTGAAGAGTAACGCA
RT-qAtACS11-R	
RT-qAtCYCB1:	CATCGTTTGGTCCGACATATTC
1-F	CGAGACGCCCCCACTACTTAGACTT
RT-qAtCYCB1:	CGGGTTTAGCTCGAATCGGACATGC
1-R	
RT-qAtUBQ5-F	GTGGTGCTAAGAAGAGGAAGA
RT-qAtUBQ5-R	TCAAGCTTCAACTCCTTCTT
RT-qAtACTIN-F	ACAGTGTCTGGATCGGTGGTTC
RT-qAtACTIN-	TGCCTCATCATACTCAGCCTTG
R	TGATCCAGAGGTGTAAAGTGAC
RT-qAT1G5168	AAGTTCTTTACCAAGAGGAGCA
0 -F	GAAGTCTTTAATCGCCGTCTTC
RT-qAT1G5168	TGAGATCATCTCCGACTTGAAG
0 -R	TCCAGTTATCATAGCAGACGAC
RT-qAT2G3049	TGGTATTGAGGTTAGGATCGTG
0 -F	GAGATCTTCCGCACAAGTTAAC
RT-qAT2G3049	CAATGGCTTCGATACCTTCAAG
0 -R	CATAACCCGCATGTTAACGTAG
RT-q	TTAACCTTCGCAACTACACTCT
AT1G27440 -F	TGTCCGATTCTCTCTAACATCG
RT-q	GGCATTGAGTTTTCTGTTCACT
AT1G27440 -R	CAAAATCATTTTCATCGACGCG
RT-q	CATCAGAAGCTGAAACGTACAG

AT2G20610 -F	TATGAGAAATGGTGCTGAGAGG
RT-q	CCAACGTTTGTTTGCTAGTACA
AT2G20610-R	GCTGGAACTACAAACAAGACTG
RT-q	GCAGGTAACATTCCATGTCATC
AT2G25450 -F	GACCTATCTAGATCGGCTTTCC
RT-q	ACTGACAAAGACATCTACAGGG
AT2G25450 -R	TAAGGCTATCTGTTCGGACTTC
RT-qAT2G3471	GCAACAGCTTGTTCATAACTCA
0 -F	CATGTTTGATGTCGATGAGCTT
RT-qAT2G3471	ATTCCGTAGTTGAGGCTTTGTA
0 -R	GATGAATTTGTTCCAGAGAGCG
RT-q	AACACTAGGATCAGCCGTTTTA
AT3G18660 -F	TTGCTGAACAAGTTATGGAAGC
RT-q	CTGCTGCAGAGTCATTCTACTA
AT3G18660 -R	AATATGCATTAGCTCAAGGTGC
RT-q	GCACGATGCTTTACCTTGATAG
AT3G49680 -F	GTTGCAGCAAACAGAAGAAGTA
RT-q	AAAATCGATTCTTGACGTTGGG
AT3G49680 -R	TGTTTCACATTTGTAGGGGAGT
RT-q	ATTTCCGATCTCTTGGGCTAAA
AT5G15630 -F	ATTTTGATGCAGTCAGTGGATG
RT-q	GCAAGCAGATTCTAGTCTTTCG
AT5G15630 -R	GTTGCCCTTATGATGCTTATGG
RT-qAT5G1742	TTCTTGAGCTCTCCGTTACATT
0 -F	CGCTTCTCATTGGTTTAATCGT
RT-q	CAGTAACAACTGAAGCGACAAA
AT5G17420 -R	GGATATTTGGTCAATCTGGCAC
RT-q	TCTAGCATTGTACCAGTCGATC
AT5G60690 -F	TATCACAACAACTGAGAGCCTT
RT-q	GCTTTCTTCGATATCAAGCGTT
AT5G60690-R	CTTGCATTTGCTTGGATGTTTG
RT-qAtDREB2A	CCAACAGCTTGTTTGATAGTCC
-F	AAGAAACAGCGTACGATGAAAC
RT-qAtDREB2A	GATTCCCTGGATCTAAAAACGC
-R	ATGGTCTCGGATTTTAGGTCTC
RT-qAtNCED3-	AGAAACTGATCTTGCGTAGTCA
F	TGAAAACAACTTCTTTAGCGGG
RT-qAtNCED3-	GACAACCACTGACTAACACAAC
R	GTTACATCTGATGTTCGGCTTC
RT-qAtABA1-F	CGTGAACAGTCAATATCAGCAG
RT-qAt ABA1-R	CTTGTGCTGAAGTCAATTCACA
RT-qAt ABA3-F	TTGGCATGATGGGTATATCCTC
RT-qAt ABA3-R	GAGGACATTCGTTTGGGTTTAC
RT-qAtWRKY4	GAAGCAATGACTGATGTAGCTG
0-F	TCACTTCCAGCAAATTTCAACC
RT-qAtWRKY4	CGTACACAGAGGAGAGAAACTT
0-R	GCTTCAAATTCAGCACTTCTCA
RT-qAt RD26-F	GTTGAAGTTGCTGGAGAGATTG
RT-qAt RD26-R	GACTTGATCACGACGTTTGTAG
RT-qAt SOS1-F	GATAAACTCCATCGTAAACGCC
RT-qAt SOS1-R	TTCCATCTAGACGACGATGTTT
RT-qAt NHX1-F	GAGGTCGTATTTGAGTTTGACG
RT-qAt NHX1-R	CAAGCATATGCATCTACGATGG
RT-qAt NHX5-F	AACTATTCTCAGCTAGACTCGC
RT-qAt NHX5-R	ATCCCCCGGGCTGCAGGAATTCATGGTGAAACAA
RT-qAt NHX6-F	GAACGCA
RT-qAt NHX6-R	CGATAAGCTTGATATCGAATTCTTAATTGAAACTC
RT-qAt TPC1-F	CAAAGCGGAATG
RT-qAt TPC1-R	
RT-qAt	

NRAMP4-F	
RT-qAt NRAMP4-R	
RT-qAt NRAMP3-F	
RT-qAt NRAMP3-R	
RT-qAt HMA3-F	
RT-qAt HMA3-R	
RT-qAt HMA4-F	
RT-qAt HMA4-R	
RT-qAt CAD1-F	
RT-qAt CAD1-R	
RT-qAt GSH1-F	
RT-qAt GSH1-R	
RT-qAt GSH2-F	
RT-qAt GSH2-R	
RT-qAt PCS2-F	
RT-qAtPCS2-R	
RT-qAt IRT1-F	
RT-qAt IRT1-R	
RT-qAt NIP1;1-F	
RT-qAt NIP1;1-R	
RT-qAt NIP3;1-F	
RT-qAt NIP3;1-R	
RT-qAt MRP1-F	
RT-qAt MRP1-R	
GAL4DB-ERF012-F	
GAL4DB-ERF012-R	
OE-ERF012-F	TCCCCCCGGGATGGTGAAACAAGAACGCAAGATCC
OE-ERF012-R	CCGCTCGAGTTAATTGAAACTCCAAAGCGGAATGT
pERF012-F	ggtcgacggatccccGTCATTTGTTGGGAACTGGTACGGA
pERF012-R	AGGGACTGACCACCCTGTGTACGTACAGGCTTTGTAGAGTG
AT1G51680-his2-F	atagggcgaattcccGTTGAAGGATGAGTTTGGTGAAGGCT
AT1G51680-his2-R	acgcgtgagctccccAGGCTTTGGCCTGAAGGAAACA
AT2G30490-his2-F	atagggcgaattcccGACCAACGAAATTCGGCATACGTG
AT2G30490-his2-R	acgcgtgagctccccGAAATGAGGACCAACGGCAAAAGG
ERF012-rec2-F	TCCCCCCGGGATGGTGAAACAAGAACGCAAGATCC
ERF012-rec2-R	CCGCTCGAGTTAATTGAAACTCCAAAGCGGAATGT
ERF012-DT2-R0	ATATATGGTCTCGATTGGTTGAGGAGAGTAATTGAAGTT
ERF012-DT1-F0	TGGTTGAGGAGAGTAATTGAAGTTTTAGAGCTAGAAATAGC
ERF012-DT2-R0	AACCGTCTTCGTCTTCGTGTAACAATCTCTTAGTCGACTCTAC
ERF012-DT2-BsR	AACCGTCTTCGTCTTCGTGTAACAATCTCTTAGTC

GACTCTAC

Use	Primers (5'->3')	
	ERF18-F	TCCCCCCGGGATGGTGAAGCAAGCGATGAAGGAAGA
	ERF18-R	CCGCTCGAGCGCGCTCAAAAATCCCAAAGAATCAAAGA
Transgenic plants	ERF12-F	TCCCCCCGGGATGGTGAAACAAGAACGCAAGATCC
	ERF12-R	CCGCTCGAGTTAATTGAAACTCCAAAGCGGAATGT
	ERF18-F	TCCCCCCGGGATGGTGAAGCAAGCGATGAAGGAAGA
	ERF18-R	CCGCTCGAGCGCGCTCAAAAATCCCAAAGAATCAAAGA
	A-AOCX3-F	TCCCCCCGGGAGTTGCTGATAAAAAAAAAAAGAGTGG
	A-AOC3-R	CCGCTCGAGCTTGGTCGGTTCGGTTGTGTCAATTTG
	C-AOC1-F	TCCCCCCGGGTTCATCTAACAAAACTATTATC
	C-AOC1-R	CCGCTCGAG GAGTTTTACGAAATGTCTATGTG
Yeast One Hybrid	ERF12-F	TCCCCCCGGGATGGTGAAACAAGAACGCAAGATCC
	ERF12-R	CCGCTCGAGTTAATTGAAACTCCAAAGCGGAATGT
	ERF18-F	CCGGAATTCATGGTGAAGCAAGCGATGAAGGAAGA
	X2-ERF18-R	GCTCTAGAAAAATCCCAAAGAATCAAAGA
	121-AOC1-F	CTCAAGATCAGAAGTATGTGGCAATGAGGTTTAGAATGG
	121-AOC1-R	AGGGACTGACCACCCAGATGGGACTTTGGTTTTATGTGG
	121-OPR3-F	CTCAAGATCAGAAGTAGTAACTCAGGGCTCACAAATGG
	121-OPR3-R	AGGGACTGACCACCCGTTGTGTGGTCGTGGAAATGGA
Transient Expression Assays	121-AOS-F	CTCAAGATCAGAAGTACGAACGCTTGGGACGTGTTTT
	121-AOS-R	AGGGACTGACCACCCACAGTGGCGAGTGTTGTGATTG
	121-AOC3-F	CTCAAGATCAGAAGTTCGTTTGGACGTTGGTA

121-AOC3-R	CAC<br>AGGGACTGACCACCCCCACTCGGTTTCGAATT<br>GTCT
